# Knowledge, attitudes, and practices regarding mycoplasma pneumoniae pneumonia among family members of affected children

**DOI:** 10.1038/s41598-026-55543-4

**Published:** 2026-05-29

**Authors:** Can Ji, Jingyi Wang, Ping Zhang, Lijian Geng, Xueping Dong

**Affiliations:** Department of Pediatrics, Xuzhou First People’s Hospital, Xuzhou, 221112 China

**Keywords:** Mycoplasma pneumoniae, Family member, Knowledge, Attitude, Practice, Cross-sectional study, Diseases, Health care, Medical research

## Abstract

This study aimed to assess the knowledge, attitudes, and practices (KAP) among family members of children with Mycoplasma pneumoniae pneumonia (MPP) and explore the relationships between these dimensions. A cross-sectional survey was conducted at Xuzhou First People’s Hospital (June-July 2025). A structured questionnaire collected demographic information and KAP data from 449 family caregivers. Adequate KAP levels were defined as scores ≥ 70% of the maximum. Structural equation modeling (SEM) analyzed the direct and indirect associations between knowledge, attitude, and practice. Most respondents were female (74.16%), with a mean age of 35.60 ± 6.62 years. The mean scores were 12.61 ± 5.00 (knowledge, range 0–20), 56.00 ± 5.74 (attitude, range 13–65), and 39.92 ± 4.63 (practice, range 9–45). Using the ≥ 70% cutoff, 170/449 (37.86%) had adequate knowledge, 442/449 (98.44%) had a positive attitude, and 430/449 (95.77%) reported proactive practices, though the latter two figures likely reflect the engagement of caregivers already active in hospital follow-up systems. In the latent-variable SEM, higher knowledge scores were positively associated with more favorable attitudes (standardized β = 0.404, *P* < 0.001), and more favorable attitudes were associated with better reported practices (standardized β = 0.570, *P* < 0.001). Knowledge showed a significant indirect association with practice through attitude (standardized β = 0.230, *P* < 0.01). In this hospital-linked sample of family caregivers (recruited from inpatient/outpatient services and WeChat follow-up groups), knowledge was limited and attitudes were generally positive, while reported practices were overall high, which may reflect selection effects. These findings and the observed associations among knowledge, attitude, and practice should be interpreted in the context of caregivers already engaged in clinical follow-up, and may not generalize to all family caregivers of children with MPP in the community.

**Supplementary Information:**

The online version contains supplementary material available at 10.1038/s41598-026-55543-4.

## Introduction

Mycoplasma pneumoniae pneumonia (MPP) has been a significant cause of community-acquired pneumonia in children worldwide in recent years, accounting for 20–40% of pediatric community-acquired pneumonia cases, with even higher rates reported during epidemic seasons and among older children^1^. In China, recent surveillance has suggested a substantial burden; data from Wuhan during 2020–2022 reported that M. pneumoniae infections accounted for 40–50% of community-acquired pneumonia cases^2^. In recent years, the increasing prevalence of antimicrobial resistance has complicated the clinical management of MPP. If the treatment is improper, the illness may persist for a long time and even lead to serious complications such as atelectasis, pleural effusion, respiratory failure, and extrapulmonary manifestations including myocarditis and encephalitis^3^.

Family members play a critical role in the early recognition and management of pediatric MPP. Family caregivers are instrumental in early detection and initiation of care for pediatric respiratory infections^4^. Previous studies have shown that family caregivers, particularly mothers, often possess insufficient knowledge regarding the etiology and clinical manifestations of pneumonia. As a result, many misinterpret pneumonia as a common cold and resort to traditional remedies to manage isolated symptoms such as cough or fever, which may contribute to delays in seeking appropriate medical care^5–7^.

The knowledge, attitudes, and practices (KAP) framework is a widely used approach in health research to assess individuals’ understanding of health issues, their beliefs and perceptions, and their actual behaviors related to health management^8^. As primary caregivers, family members’ KAP may be associated with healthcare-seeking behavior, antibiotic adherence, and complication recognition, all of which are relevant to the child’s clinical management and outcomes^4,9^. However, most existing KAP investigations focus on general pediatric pneumonia^4,10^. A cross‑sectional survey on pediatric pneumonia in China noted favorable attitudes and practices among parents but significant knowledge gaps regarding etiology and prevention^10^. These findings highlight the necessity for disease-specific KAP assessment, especially for MPP, given its unique clinical trajectories and potential for outbreaks. To the best of our knowledge, limited studies have specifically investigated KAP regarding MPP, particularly among family caregivers—a population that plays a critical role in disease management. Given the outbreak-prone nature of MPP and the caregiver-relevant challenges in home management (e.g., recognizing complications, antibiotic-related concerns, follow-up, and infection control), a disease-specific caregiver KAP assessment is needed to identify actionable targets for education.

Therefore, this study had three objectives: (1) to describe caregivers’ knowledge, attitudes, and practices regarding MPP and estimate the prevalence of adequate KAP using the prespecified ≥ 70% cutoff; (2) to examine correlations among KAP domain scores; and (3) to test the hypothesized mediation structure in which attitude mediates the association between knowledge and practice using structural equation modeling (SEM).

## Methods

### Study design and participants

A cross-sectional study was conducted between June and July 2025 at Xuzhou First People’s Hospital, involving family members of children diagnosed with MPP. Participants were recruited through a combination of inpatient wards, outpatient clinics, and supervised online distribution. Specifically, family members of children diagnosed with MPP were identified from two sources: (1) hospitalized patients with laboratory-confirmed M. pneumoniae infection in the pediatric wards, and (2) outpatients with positive M. pneumoniae test results in the outpatient clinics. In addition, the questionnaire link was distributed via WeChat follow-up groups that were established for patients and families who had been diagnosed and were receiving medical care or follow-up in the hospital or affiliated community clinics. Therefore, online distribution was restricted to hospital- and community-based follow-up groups and did not target the general public. The study was carried out after the protocol was approved by the Medical Ethics Committee of Xuzhou First People’s Hospital (No. xyyll [2025]101), and informed consent was obtained from all participants prior to data collection. Participants were eligible for inclusion if they met the following criteria: (1) they were family members of children with a confirmed diagnosis of MPP^11^; (2) they were aged 18 years or older; and (3) they were able to comprehend the study objectives and voluntarily provided informed consent. No additional clinical exclusion criteria were applied beyond the eligibility criteria; however, responses failing predefined data-quality checks were excluded prior to analysis, as described below.

## Questionnaire

The questionnaire was developed with reference to established literature^4,10^ and relevant clinical guidelines, including Evidence‑based guideline for the diagnosis and treatment of Mycoplasma pneumoniae pneumonia in children (2023)^12^ and Guidelines for the Diagnosis and Treatment of Mycoplasma pneumoniae Pneumonia in Children (2023 Edition)^11^. Following the completion of the initial draft, content validity was supported through expert review to evaluate item relevance and clarity. Three experts, each with over 30 years of experience in pediatrics, were invited to review the questionnaire, and appropriate modifications were made based on their feedback. A pilot test was subsequently carried out with a sample of 42 participants to assess the reliability of the instrument. The results demonstrated high internal consistency, with an overall Cronbach’s α coefficient of 0.927. Specifically, the Cronbach’s α values were 0.914 for the knowledge dimension, 0.886 for the attitude dimension, and 0.829 for the practice dimension, indicating satisfactory reliability across all components of the questionnaire. In addition, construct validity of the instrument was examined using Kaiser–Meyer–Olkin (KMO) testing and confirmatory factor analysis (CFA). The KMO value was 0.919 (*P* < 0.001), and the CFA model demonstrated acceptable fit (CMIN/DF = 2.334, RMSEA = 0.055, IFI = 0.927, TLI = 0.918, CFI = 0.926), with statistically significant item loadings on their intended latent constructs (**Tables S1-2**).

The finalized instrument, administered in Chinese, comprised four sections: demographic information, knowledge, attitude, and practice. The demographic section collected information on both the child (age, gender, time of diagnosis, and history of MPP) and the participating family member (age, gender, place of residence, educational level, marital status, relationship to the child, monthly per capita household income, history of MPP, and sources of health education). The knowledge section consisted of 10 items, each rated on a three-point scale: “Very familiar” (2 points), “Heard of it” (1 point), and “Unclear” (0 points), yielding a total possible score ranging from 0 to 20. The attitude section included 13 items evaluated using a five-point Likert scale, with responses scored from “Strongly agree” (5 points) to “Strongly disagree” (1 point), resulting in a total score range of 13 to 65. The practice section comprised 9 items, also measured on a five-point scale, with response options from “Never” (1 point) to “Always” (5 points), giving a total score range of 9 to 45. For interpretative purposes, a score equal to or exceeding 70.0% of the maximum possible score in each respective dimension was considered indicative of adequate knowledge, a positive attitude, and proactive behavioral practices based on previous KAP studies^13,14^. We selected the 70% threshold because it has been commonly applied in prior KAP studies to provide a pragmatic and comparable definition of “adequate” performance; however, we acknowledge that this cut-off is empirical rather than a clinically validated standard.

## Questionnaire distribution and quality control

Questionnaires in this study were administered through an online platform. Before formal distribution of the questionnaire link, all potential participants were provided with a brief explanation of the study objectives, procedures, and voluntary nature of participation to support informed consent and eligibility confirmation. To minimize duplicate responses, participants were instructed to complete the questionnaire only once, and the online survey platform was configured to restrict multiple submissions from the same account or device where technically feasible.This online survey was created using the “Wenjuanxing” platform (https://www.wjx.cn/) and a corresponding QR code was generated for distribution. The QR code was shared via WeChat with eligible participants in hospital wards and outpatient clinics. Additionally, a portion of the questionnaires was distributed through family communication groups for MPP, in order to facilitate data collection. To ensure the integrity of the data and enhance the credibility of the study findings, a series of exclusion criteria were applied to identify and remove responses exhibiting potential information bias or selection bias. First, a validation (trap) question - “The capital of China is Shanghai”—was embedded within the questionnaire to detect inattentive respondents; those who answered this item incorrectly were excluded from analysis. Second, questionnaires completed in less than 60 s were considered invalid, as such a brief completion time was insufficient for careful reading and thoughtful responses. Third, entries containing implausible or extreme values, such as an age of 150 years, were removed to minimize the influence of systematic errors and outliers on statistical estimates. Lastly, submissions displaying uniform response patterns—such as selecting the same option across all items—were excluded, as such patterns are indicative of careless or insincere participation. For missing data, all KAP items and key demographic variables were set as mandatory responses in the questionnaire, so there were no missing data in the analyzed dataset.

## Sample size

The required sample size for this study was calculated based on a previously published methodology^15^, using the formula n = (z²pq)/e², where n denotes the sample size, z is the z-score corresponding to a 95% confidence level (1.96), p is the estimated proportion, q is 1–p, and e represents the margin of error, set at 5%. To ensure a conservative estimate and maximize the sample size, an expected proportion (p) of 0.5 was used. This yielded a minimum required sample size of 384 participants. After adjusting for an anticipated 20% rate of invalid responses, the final minimum sample size was set at 480.

### Statistical analysis

Data analysis was performed using STATA version 14.0 (StataCorp, College Station, TX, USA). Continuous variables were described as means ± standard deviations (SD). The normal distribution of continuous data was assessed using the Kolmogorov-Smirnov test. For continuous variables conforming to a normal distribution, Student’s t-test or ANOVA was used for group comparisons. Those with a skewed distribution were analyzed using the Wilcoxon-Mann-Whitney U-test or the Kruskal-Wallis analysis of variance. Categorical variables were presented as n (%). Spearman’s correlation was employed to determine the correlation among the KAP scores. Based on the knowledge-attitude-practice theoretical model, SEM was applied to assess the mediating effect of attitude in the association between knowledge and practice. A latent-variable SEM was specified, in which knowledge, attitude, and practice were modeled as latent constructs measured by their respective questionnaire items (K1–K10, A1–A13, and P1–P9). For model identification, the loading of the first indicator in each domain (K1, A1, and P1) was fixed to 1. Direct and indirect effects were estimated and compared. Bias-corrected bootstrap with 5,000 resamples was used to estimate confidence intervals and P-values for indirect and direct effects. Path-coefficient P-values reported for the main structural paths were based on critical ratio (CR) tests, whereas bootstrap-based P-values were used for the decomposition of standardized total, direct, and indirect effects. Model fit was evaluated using the following indices: root mean square error of approximation (RMSEA) < 0.08, standardized root mean square residual (SRMR) < 0.08, Tucker–Lewis index (TLI) > 0.80, and comparative fit index (CFI) > 0.80. To assess the robustness of our categorical interpretations, a sensitivity analysis was pre-specified using an alternative, more stringent threshold of 80% of the maximum possible score to define adequacy for each KAP dimension. All statistical tests were two-sided, and a P-value of less than 0.05 was considered statistically significant.

## Results

Initially, a total of 509 participants were enrolled. Five declined to participate, leaving 504 respondents. After applying exclusion criteria, the following cases were removed: 3 questionnaires with completion time < 60s; 14 family members under 18 years of age and 2 with abnormal values for age; 12 children aged ≥ 18 years; 24 questionnaires with incorrect answers to the trap question (**Figure ****S1**). The final valid sample included 449 participants, with a valid response rate of 88.21%. Participant characteristics are presented in Table 1.

**Table 1:** Demographic characteristics and KAP scores of participants (*N* = 449).

*N* = 449	*N* (%)	Knowledge score		Attitude score		Practice score	
		Mean ± SD	*P*	Mean ± SD	*P*	Mean ± SD	*P*
**Total score**		12.61 ± 5.00		56.00 ± 5.74		39.92 ± 4.63	
**Gender**			0.170		0.436		0.769
Male	116(25.84)	11.97 ± 5.39		55.76 ± 5.63		39.75 ± 4.98	
Female	333(74.16)	12.82 ± 4.84		56.07 ± 5.78		39.97 ± 4.51	
**Age (years)**	35.60 ± 6.62		0.067		< 0.001		0.005
< 35	220(49.00)	12.99 ± 5.02		57.15 ± 5.21		40.63 ± 4.00	
≥ 35	229(51.00)	12.23 ± 4.96		54.87 ± 6.01		39.23 ± 5.08	
**Residence**			0.002		0.232		0.036
Rural	75(16.7)	10.98 ± 4.74		55.09 ± 5.97		38.82 ± 5.04	
Urban	374(83.3)	12.93 ± 4.99		56.17 ± 5.68		40.13 ± 4.52	
**Education**			< 0.001		0.071		0.002
High school/Technical secondary school or below	80(17.82)	10.52 ± 4.47		54.87 ± 6.31		38.65 ± 6.04	
Associate degree	109(24.28)	12.43 ± 4.82		56.88 ± 5.58		40.32 ± 4.12	
Bachelor’s degree	209(46.55)	13.24 ± 4.94		56.22 ± 5.76		40.53 ± 4.29	
Master’s degree or above	51(11.36)	13.60 ± 5.57		54.94 ± 4.66		38.52 ± 3.85	
**Marital status**			0.369		0.239		0.587
Other	17(3.79)	11.17 ± 5.60		54.64 ± 4.79		40.76 ± 3.54	
Married	432(96.21)	12.66 ± 4.97		56.04 ± 5.77		39.88 ± 4.67	
**Relationship with the child**			0.202		0.585		0.440
Father/Mother	421(93.76)	12.67 ± 4.97		56.01 ± 5.77		39.87 ± 4.65	
Other	28(6.24)	11.53 ± 5.44		55.71 ± 5.42		40.53 ± 4.32	
**Monthly income per capita (CNY)**			0.001		0.821		0.085
< 2000	38(8.46)	10.84 ± 3.96		55.68 ± 6.52		38.92 ± 5.81	
2000–5000	130(28.95)	11.76 ± 4.72		55.80 ± 6.19		39.41 ± 5.16	
5000–10,000	191(42.54)	13.27 ± 5.07		56.33 ± 5.43		40.62 ± 4.05	
> 10,000	90(20.04)	13.14 ± 5.33		55.67 ± 5.39		39.57 ± 4.27	
**Had mycoplasma pneumonia**			0.008		0.901		0.649
Yes	162(36.08)	13.46 ± 4.63		55.92 ± 5.84		39.87 ± 4.39	
No	287(63.92)	12.11 ± 5.14		56.03 ± 5.69		39.94 ± 4.76	
**Educational information about mycoplasma pneumonia**			< 0.001		0.153		0.003
Yes	251(55.9)	14.06 ± 4.68		56.37 ± 5.69		40.41 ± 4.59	
No	198(44.1)	10.75 ± 4.78		55.51 ± 5.78		39.28 ± 4.61	
**Child’s gender**:			0.829		0.010		0.333
Male	252(56.12)	12.45 ± 5.17		55.36 ± 6.05		39.66 ± 4.94	
Female	197(43.88)	12.80 ± 4.77		56.79 ± 5.23		40.24 ± 4.19	
**Child’s age**	5.58 ± 3.68		0.958		0.166		0.010
≤ 5 years	248(55.23)	12.58 ± 5.05		56.37 ± 5.41		40.43 ± 4.28	
> 5 years	201(44.77)	12.63 ± 4.95		55.53 ± 6.10		39.27 ± 4.96	
**Time since diagnosis**			0.111		0.408		0.172
Within a month	225(50.11)	12.12 ± 5.09		55.96 ± 5.74		40.11 ± 4.76	
1–3 months	19(4.23)	12.68 ± 4.29		54.94 ± 6.13		39.15 ± 5.79	
3–6 months	21(4.68)	14.57 ± 5.40		57.90 ± 5.99		41.61 ± 3.85	
6–12 months	39(8.69)	12.30 ± 4.09		57.02 ± 5.37		40.15 ± 4.02	
Over 1 year	145(32.29)	13.13 ± 5.04		55.63 ± 5.73		39.41 ± 4.48	
**MPP history of the child**			< 0.001		0.176		0.961
Yes	278(61.92)	13.65 ± 4.59		56.26 ± 5.88		39.94 ± 4.52	
No	171(38.08)	10.90 ± 5.17		55.54 ± 5.50		39.88 ± 4.82	

## Participant characteristics

The study included 449 family members of children with MPP. The majority of family members were female (74.16%), married (96.21%), and resided in urban areas (83.3%). The mean age was 35.60 ± 6.62 years, with 51.00% aged ≥ 35 years. Most held a bachelor’s degree or higher (57.91%) and had a monthly per capita income ≥ 5,000 CNY (62.58%). Parents accounted for 93.76% of the respondents, and 36.08% had a personal history of MPP. Among the children, 56.12% were male, with a mean age of 5.58 ± 3.68 years; 55.23% were aged ≤ 5 years. Over half (61.92%) had a history of MPP, and 50.11% had been diagnosed within the past month **(**Table 1**)**. When it comes to their sources of information about mycoplasma pneumonia, hospital promotions and doctors’ health education were reported by 75.72%, followed by new media (68.37%), and communication with relatives, friends or patients (54.12%).

## Overall KAP scores and adequacy prevalence

Their mean knowledge, attitude, and practice scores were 12.61 ± 5.00 (possible range: 0–20), 56.00 ± 5.74 (possible range: 13–65), and 39.92 ± 4.63 (possible range: 9–45), respectively. Based on the ≥ 70% cutoff, 170/449 (37.86%) had adequate knowledge, 442/449 (98.44%) had a positive attitude, and 430/449 (95.77%) met the threshold for practice scores **(**Table 2**)**. To assess the robustness of these categorical findings, a sensitivity analysis was performed using a more stringent threshold of 80% of the maximum possible scores. Under this criterion, the prevalence of adequate knowledge, attitude, and practice was 123/449 (27.4%), 297/449 (66.1%), and 334/449 (74.4%), respectively, which remained broadly consistent with the primary analysis. Overall, family caregivers of children with MPP exhibited limited knowledge, generally positive attitudes, and relatively high levels of reported practices.

**Table 2:** Adequate knowledge, attitude, and practice levels based on the ≥ 70% cutoff (*N* = 449).

	Knowledge		Attitude		Practice	
	Scores	Number	Scores	Number	Scores	Number
70% cutoff						
Adequate/positive/proactive	(14,20]	170	(45,65]	442	(31,45]	430
Inadequate/non-positive/non-proactive	[0,14]	279	[34,45]	7	[13,31]	19
80% cutoff						
Adequate/positive/proactive	(16,20]	123	(52,65]	297	(36,45]	334
Inadequate/non-positive/non-proactive	[0,16]	326	[34,52]	152	[13,36]	115

Urban residents had higher knowledge and practice scores than rural counterparts (both *P* < 0.05). Bachelor’s-educated participants outperformed those with ≤high school in knowledge (*P* < 0.001) and practice (*P* = 0.002). Prior MPP infection in families (*P* = 0.008) and receipt of MPP-related educational information (*P* < 0.001) were associated with higher knowledge scores, while receipt of educational information was also associated with higher practice scores (*P* = 0.003). Caregivers < 35 years showed better attitudes (*P* < 0.001) and practices (*P* = 0.005) than older peers. Additionally, households with children ≤ 5 years scored higher in practices (*P* = 0.010), and those with monthly income 5,000–10,000 CNY scored higher in knowledge (*P* = 0.001). Female caregivers of girls had slightly higher attitude scores (*P* = 0.010) (Table 1).

### Item-level highlights

Although overall attitude and practice adequacy rates were high, item-level responses suggested variability across specific behaviors **(Tables S3–S5)**. The distribution of knowledge dimensions showed that the three questions with the highest number of participants choosing the ‘Unclear’ option were ‘About one-quarter of children with mycoplasma pneumonia have extrapulmonary manifestations, which may include myocarditis, arrhythmia, encephalitis, and kidney disease.’ (K6) with 28.29%, ‘Pulmonary complications of mycoplasma pneumonia may include atelectasis, pleural effusion, and respiratory failure.’ (K5) with 23.16%, and ‘Mycoplasma pneumonia is a self-limiting disease, and most children have a good prognosis after proper treatment.’ (K8) with 13.36% (**Table ****S3**). In contrast, uncertainty was low for basic commonality and prevention items, with only 5.12% selecting the Unclear option for K1 and K10, suggesting that knowledge gaps were domain-specific rather than uniformly low.

Overall, attitudes were generally positive across items related to timely treatment, adherence, prevention, and willingness to learn. Compared to other common types of pneumonia, 33.85% strongly agreed that they feel more anxious when their child has mycoplasma pneumonia (A2). Only 28.95% reported being very willing to use non-pharmacological methods to support recovery (A9) (**Table S4**).

Practice items suggested stronger endorsement of adherence-type behaviors (e.g., P2 and P3), whereas continuity and prevention behaviors were less consistently reported. Responses to the practice dimension showed that only 40.53% always take their child to see a doctor immediately when symptoms of cough and fever appear (P1), only 45.21% always take their child for a follow-up check to ensure complete recovery (P8), only 46.33% always clean and disinfect the home environment to prevent reinfection with mycoplasma pneumonia (P7) (**Table S5**).

### Correlations between KAP

Further correlation analysis revealed positive correlations between knowledge scores and attitude scores (*r* = 0.3545, *P* < 0.001), as well as between knowledge scores and practice scores (*r* = 0.3245, *P* < 0.001). Additionally, attitude scores were positively correlated with practice scores (*r* = 0.5339, *P* < 0.001) (Table 3).

**Table 3:** Correlations between knowledge, attitude, and practice scores.

	Knowledge	Attitude	Practice
Knowledge	1		
**Attitude**	0.3545 (*P*<0.001)	1	
**Practice**	0.3245 (*P*<0.001)	0.5339 (*P*<0.001)	1

### Mediation analysis

**Fig. 1:**
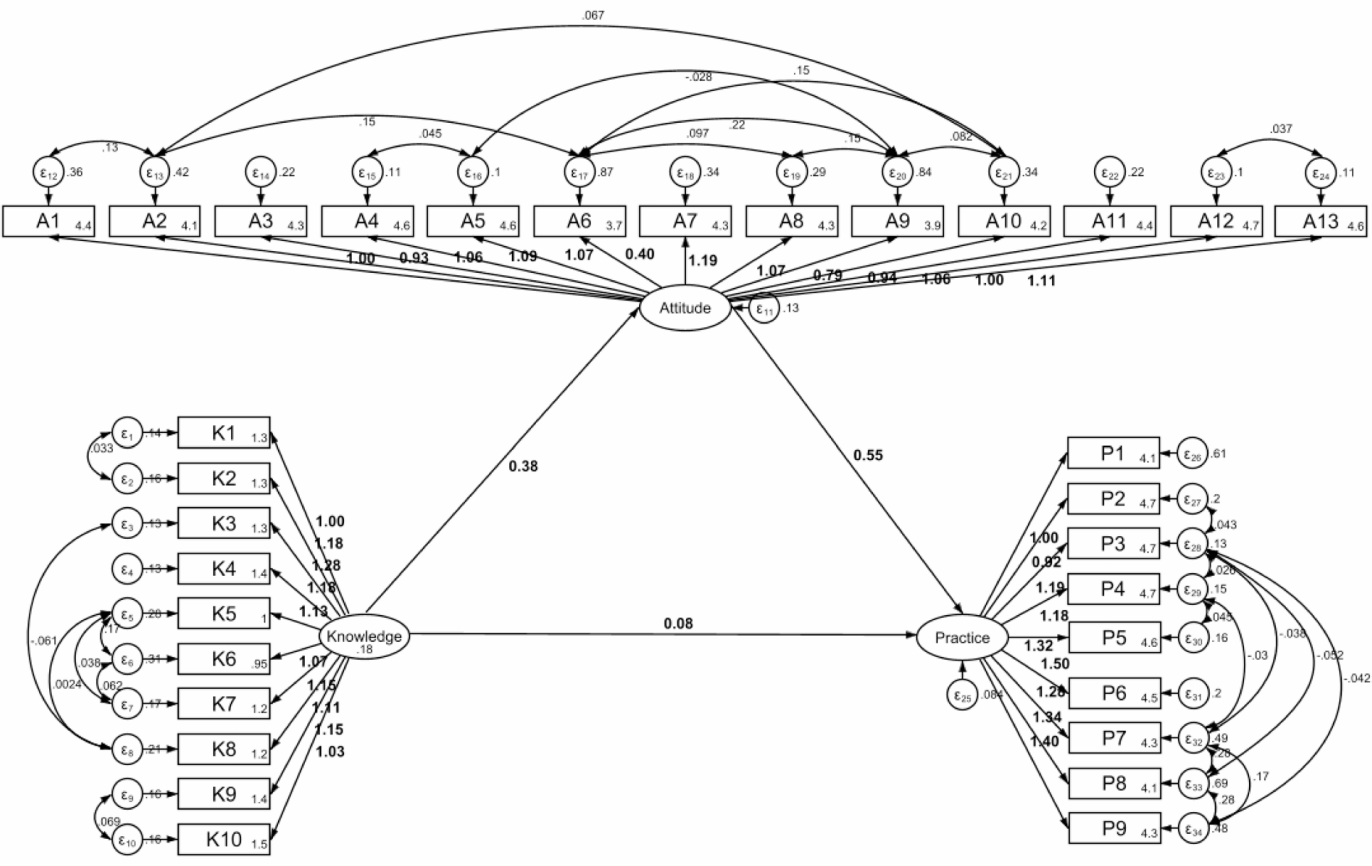
Structural equation model with latent constructs for knowledge, attitude, and practice, showing the associations among the three domains.Ellipses represent latent variables (unobserved constructs), including overall knowledge, attitude, and practice. Rectangles represent observed variables (measured questionnaire items) that serve as indicators of the latent variables. Circles represent error terms or residuals associated with the observed variables. Single-headed arrows indicate hypothesized directional relationships (pathways) between latent variables and from latent variables to observed variables, with standardized path coefficients labeled on the arrows.

The SEM demonstrated good model fit (RMSEA = 0.051; SRMR = 0.052; TLI = 0.928; CFI = 0.937) **(Table S6)**. The model indicated that knowledge was positively associated with attitude (standardized β = 0.404, P < 0.001), and attitude was positively associated with practice (standardized β = 0.570, P < 0.001). Notably, the latent ‘Practice’ construct was predominantly associated with medication and treatment-adherence behaviors (P2–P6; standardized loadings 0.583–0.820), whereas early care-seeking (P1; loading 0.390), home disinfection (P7; loading 0.507), follow-up after recovery (P8; loading 0.475), and P9 (loading 0.553), indicating that the attitude–practice pathway primarily reflects adherence-type behaviors. Knowledge was also indirectly associated with practice through attitude (standardized β = 0.230, *P* < 0.01) **(**Table 4 and Fig. 1**).** The direct path from knowledge to practice was not statistically significant (standardized β = 0.088, *P* = 0.079), whereas the total effect remained significant (standardized β = 0.318, *P* = 0.016). Taken together, these results indicate that the association between knowledge and practice was primarily indirect via attitude (standardized indirect effect β = 0.230, *P* = 0.004), rather than a direct association. The measurement model estimates indicated that the observed items loaded significantly on their intended latent constructs **(**Table 5**).**

**Table 4:** Standardized total, direct, and indirect effects in the structural equation model.

Model paths	Standardized total effects		Standardized direct effects		Standardized indirect effects	
	β (95% CI)	*P*	β (95% CI)	*P*	β (95% CI)	*P*
Knowledge →Attitude	0.404 (0.310,0.487)	0.013	0.404 (0.310,0.487)	0.013		
Knowledge →Practice	0.318 (0.209,0.405)	0.016	0.088 (-0.011, 0.207)	0.079	0.230 (0.175,0.321)	0.004
Attitude → Practice	0.570 (0.495,0.709)	0.003	0.570 (0.495,0.709)	0.003		

Standardized total, direct, and indirect effects were estimated using the SEM model. Bias-corrected bootstrap with 5,000 resamples was used to calculate 95% confidence intervals and P-values for the decomposition of effects. Path-coefficient P-values reported in the main text and Table 5 were based on critical ratio (CR) tests under normality assumptions.

**Table 5:** Measurement model estimates for the SEM, including unstandardized and standardized factor loadings.

			Unstandardized estimate	Standardized estimate	S.E.	C.R.	P
Attitude	<---	Knowledge	0.411	0.404	0.054	7.632	<0.001
Practice	<---	Attitude	0.440	0.570	0.066	6.628	<0.001
Practice	<---	Knowledge	0.069	0.088	0.039	1.759	0.079
K1	<---	Knowledge	1.000	0.760			
K2	<---	Knowledge	1.174	0.786	0.067	17.442	<0.001
K3	<---	Knowledge	1.247	0.827	0.068	18.370	<0.001
K4	<---	Knowledge	1.156	0.812	0.064	18.123	<0.001
K5	<---	Knowledge	1.130	0.682	0.076	14.820	<0.001
K6	<---	Knowledge	1.081	0.649	0.077	14.004	<0.001
K7	<---	Knowledge	1.149	0.771	0.067	17.052	<0.001
K8	<---	Knowledge	1.087	0.711	0.071	15.373	<0.001
K9	<---	Knowledge	1.124	0.766	0.066	16.909	<0.001
K10	<---	Knowledge	1.008	0.728	0.063	15.940	<0.001
A13	<---	Attitude	1.000	0.788			
A12	<---	Attitude	0.887	0.766	0.042	21.188	<0.001
A11	<---	Attitude	0.931	0.647	0.067	13.916	<0.001
A10	<---	Attitude	0.861	0.541	0.076	11.407	<0.001
A9	<---	Attitude	0.718	0.320	0.110	6.507	<0.001
A8	<---	Attitude	0.976	0.619	0.074	13.248	<0.001
A7	<---	Attitude	1.086	0.630	0.080	13.518	<0.001
A6	<---	Attitude	0.410	0.188	0.109	3.773	<0.001
A5	<---	Attitude	0.968	0.797	0.055	17.695	<0.001
A4	<---	Attitude	0.987	0.788	0.057	17.433	<0.001
A3	<---	Attitude	0.967	0.673	0.066	14.587	<0.001
A2	<---	Attitude	0.872	0.508	0.082	10.629	<0.001
A1	<---	Attitude	0.916	0.554	0.078	11.683	<0.001
P1	<---	Practice	1.000	0.390			
P2	<---	Practice	0.977	0.583	0.117	8.373	<0.001
P3	<---	Practice	1.310	0.767	0.165	7.920	<0.001
P4	<---	Practice	1.420	0.815	0.177	8.039	<0.001
P5	<---	Practice	1.537	0.820	0.191	8.050	<0.001
P6	<---	Practice	1.593	0.753	0.202	7.881	<0.001
P7	<---	Practice	1.276	0.507	0.187	6.828	<0.001
P8	<---	Practice	1.367	0.475	0.206	6.623	<0.001
P9	<---	Practice	1.422	0.553	0.200	7.095	<0.001

values were based on the critical ratio (CR) test under normality assumptions.

## Discussion

Family members of children diagnosed with MPP demonstrated insufficient knowledge and generally positive attitudes, while reported practices were overall high but varied across domains, with lower consistency in continuity and prevention behaviors; significant interrelations were observed among all three KAP domains. These findings highlight potential targets for caregiver education.

Despite generally favorable attitudes and high reported practices, participants demonstrated limited understanding of essential aspects of MPP, particularly its pathogenesis, transmission, and potential complications. Similar patterns have been observed in Egypt, where a study of 200 non-specialized physicians treating children with acute upper respiratory tract infections found inadequate knowledge about antibiotic prescribing despite positive attitudes toward appropriate treatment^13^. Likewise, research in Pakistan during the COVID-19 pandemic revealed that while parents demonstrated proactive practices in seeking care for children’s upper respiratory infections, their understanding of appropriate antibiotic use remained limited^16^. These findings parallel our results, where family caregivers of children with MPP showed generally positive attitudes (mean score 56.00 ± 5.74) and proactive practices (mean score 39.92 ± 4.63), yet demonstrated insufficient knowledge (mean score 12.61 ± 5.00 out of possible 20). These findings suggest that although practical caregiving behaviors may be sustained through clinical advice and prior experience, foundational gaps in disease-specific knowledge remain, particularly in domains requiring understanding of microbial etiology or preventive mechanisms.

Correlation analysis and SEM further characterized the associations among KAP domains. The SEM results suggested that knowledge was not directly associated with practice after accounting for attitude, whereas the indirect association through attitude was statistically significant. Although the direct association of knowledge on practice was not statistically significant after adjustment for attitudinal mediation, the indirect pathway was significant. This suggests that caregivers’ reported practices may be more closely associated with internalized attitudes shaped by knowledge than with knowledge alone. This observation is consistent with health behavior frameworks that position attitudes as proximal correlates of action, particularly in acute pediatric illness contexts where urgency and anxiety are heightened^17^. The strength of the attitude-practice linkage also reflects the possibility that behavioral adherence is often maintained through trust in professional recommendations rather than through comprehensive understanding^18,19^. Statistical significance does not necessarily imply clinically meaningful change, and the SEM coefficients should be interpreted as modest-to-moderate associations. Because the study is cross-sectional and practice was self-reported, we cannot determine whether these effect sizes translate into measurable improvements in child health outcomes (e.g., complications or recovery), which should be tested in longitudinal or interventional studies.

Sociodemographic analysis highlighted distinct disparities in KAP scores. The results of this study indicated that urban residence and higher educational attainment were both associated with increased knowledge and better reported practices, reflecting broader structural inequities in information access and healthcare engagement. Such differences are consistent with prior caregiver-focused pneumonia research showing that residence and educational level are associated with caregiver knowledge and care-seeking or home management behaviors for pediatric respiratory infections^4–7,10,20,21^. However, the effect of postgraduate education on practice was less pronounced, which may reflect competing priorities, lower perceived risk, or reduced reliance on clinical authority among this group. Age also emerged as a relevant factor, with younger caregivers reporting more proactive attitudes and practices. These differences may be shaped by generational exposure to digital health platforms and evolving norms around pediatric health management^22,23^.

Experience with MPP and prior receipt of educational materials were both associated with higher knowledge and practice scores. This finding supports the relevance of experiential learning and clinician-delivered health education for caregiver engagement, although causal effects cannot be inferred from the present cross-sectional data. However, the fact that nearly half of the sample had not received targeted information about MPP, despite prior caregiving experience, points to missed opportunities for structured education within the healthcare system. The limited variation in attitude scores across groups may reflect a uniformly high level of concern among caregivers when facing respiratory infections in children, regardless of background. While this may support the notion of attitudes as emotionally driven and less sensitive to external stratifiers, it also suggests that the pathway from concern to competent action may falter in the absence of accessible and accurate information^24,25^.

The item-level analysis offers further insights. In the knowledge dimension, a substantial proportion of participants struggled with key concepts, particularly those involving transmission, complications, and clinical management. These knowledge gaps are not unique to this setting and have been reported in similar studies addressing common pediatric infections, where caregivers are often unaware of less overt or longer-term risks associated with the illness^26,27^. The limited awareness of extrapulmonary manifestations and misunderstanding of treatment principles suggest a need for more nuanced education strategies that extend beyond symptom recognition.

In the attitude section, responses were generally positive, but antibiotic-related concerns and lower willingness to use non-pharmacological measures suggest potential uncertainty about treatment strategies. Specifically, 58.79% of caregivers agreed or strongly agreed with concerns about antibiotics (A6), whereas antibiotic-related knowledge was assessed only broadly in K7 and did not cover antibiotic resistance or appropriate selection. This may explain why high medication adherence (P2) could coexist with limited antibiotic-specific understanding. Future studies should further examine item-level associations, such as whether antibiotic concern is linked to adherence or to potentially inappropriate behaviors, including premature discontinuation or avoidance. This issue is also consistent with broader concerns about antibiotic overuse and caregivers’ reliance on pharmacological treatment in pediatric respiratory infections^28,29^.

Practice items reflected high compliance with immediate clinical advice, including medication timing and symptom monitoring. However, behaviors requiring anticipation or sustained effort—such as environmental disinfection and follow-up visits—were reported less frequently, indicating that even in a highly motivated population, long-term disease management behaviors may not be fully integrated into routine care. In addition, responses in Table S3 suggest that care-seeking behavior (P1: seeking medical attention immediately) was more variable than home-care behaviors (e.g., medication adherence and symptom monitoring), indicating a specific gap in timely service utilization despite generally high reported practices.

These results should be viewed within the context of structural characteristics of the Chinese healthcare system. Despite ongoing reforms, preventive care and health education remain unevenly distributed across urban and rural settings. Hospital-centered outreach efforts may effectively promote acute compliance, yet fail to deliver comprehensive understanding or continuity of care outside the clinical setting. Additionally, the increasing reliance on digital platforms for health communication presents both opportunity and risk, as the quality of online information varies, and not all caregivers possess equal digital literacy^30,31^. The urban advantage observed in this study may thus reflect not only better infrastructure but also higher engagement with diversified information sources, a disparity that continues to shape health behaviors and outcomes across socioeconomic strata.

Actionable implications can be drawn from the specific deficits observed at the item level. First, education should prioritize recognition of complications and extrapulmonary manifestations, as uncertainty was highest for these items (e.g., K5–K6), and caregivers may otherwise underestimate severity or miss warning signs. Second, counselling should set clear expectations regarding the role of antibiotics and the value of supportive care, given prominent concerns about antibiotic use (A6) and lower willingness to use non-pharmacological measures (A9); brief, standardized explanations during visits may reduce misconceptions and improve adherence to the overall care plan. Third, continuity and prevention behaviors require explicit reinforcement, as early care-seeking, home disinfection, and follow-up were less consistently reported (P1, P7, P8). Practical checklists and simple reminders at discharge or follow-up contacts may help caregivers translate general concern into consistent home management behaviors.

However, any intervention implications should be considered suggestive because the data are cross-sectional and self-reported. The discrepancy between relatively low knowledge and very high reported practice may partly reflect social desirability and selection bias, as caregivers recruited from inpatient wards, outpatient clinics, and WeChat follow-up groups were likely already engaged with healthcare services. Thus, the high practice scores may not be representative of all community caregivers of children with MPP. In addition, several practice items reflected general adherence behaviors, such as medication use, temperature monitoring, rest, and diet, which can be performed according to clinician instructions without detailed MPP-specific knowledge. By contrast, behaviors requiring anticipatory planning or sustained effort, such as early care-seeking, home disinfection, and follow-up, were less consistently reported. The SEM measurement model similarly showed that the latent Practice construct was mainly driven by adherence-oriented items, whereas early care-seeking, home disinfection, and follow-up contributed less strongly. Therefore, the attitude–practice pathway should be interpreted primarily as an association with adherence/compliance-type behaviors rather than all practice domains. These findings suggest that caregiver education should not only provide instructions but also explain the rationale for warning-sign recognition, follow-up, and infection control.

Future educational strategies may also need to be tailored according to caregiver characteristics. For example, younger caregivers may be more reachable through digital platforms, whereas older or less educated caregivers may benefit from face-to-face education embedded within community health services. These strategies should be integrated into routine care workflows to support continuity between hospital-based and community-based follow-up^32,33^.

This study has several limitations. First, its cross-sectional design precludes causal inference among knowledge, attitudes, and practices. Accordingly, SEM pathways should be interpreted as hypothesized associations rather than evidence of temporal or causal relationships. Second, the single-hospital, hospital-linked recruitment strategy, including WeChat follow-up groups, may have preferentially included caregivers already engaged in clinical follow-up, potentially overestimating self-reported practices and limiting generalizability to community caregivers. Third, practices were self-reported and may be affected by recall and social desirability biases; data-quality filters may also have introduced selection effects. Fourth, antibiotic-related knowledge was assessed only broadly, and item-level associations, such as antibiotic concern and medication adherence, were not examined. Finally, although internal consistency was high, further validation is needed in independent and more diverse samples, particularly because several indicators showed low factor loadings and the empirical ≥ 70% adequacy cut-off was used mainly for descriptive purposes.

## Conclusion

In conclusion, in this hospital-linked sample of family caregivers of children diagnosed with MPP, knowledge was insufficient and attitudes were generally positive, while reported practices were overall high but less consistent for continuity and prevention behaviors relevant to home management. These cross-sectional findings may inform the content priorities of caregiver education (e.g., complications/extrapulmonary features, antibiotic and supportive care expectations, and follow-up, disinfection, and early care-seeking behaviors), while the effectiveness of such approaches requires confirmation in longitudinal or interventional studies.

## Supplementary Information

Below is the link to the electronic supplementary material.

Supplementary Material 1

Supplementary Material 2

Supplementary Material 3

## Data Availability

All data generated or analysed during this study are included in this published article.

## Abbreviations

KAP: Knowledge, attitudes, and practices
MPP: Mycoplasma pneumoniae pneumonia
SEM: Structural equation modeling
KMO: Kaiser–Meyer–Olkin
RMSEA: Root mean square error of approximation
SRMR: Standardized root mean square residual
TLI: Tucker–Lewis index
CFI: Comparative fit index
IFI: Incremental fit index
SD: Standard deviation
ANOVA: Analysis of variance
CI: Confidence interval
CR: Critical ratio
