# Supplementary material for: Knowledge, attitudes, and practices regarding mycoplasma pneumoniae pneumonia among family members of affected children
Source: Sci Rep. 2026 May 29;16:24617. doi: 10.1038/s41598-026-55543-4 (PMC13454310; doi:10.1038/s41598-026-55543-4)
Supplement: Supplementary file 1 [file 41598_2026_55543_MOESM1_ESM.docx]

Questionnaire ID:

Dear Participant,

We are researchers from the hospital and sincerely invite you to participate in our study. This research aims to assess the knowledge, attitudes, and practices of family members of children diagnosed with Mycoplasma pneumoniae pneumonia. The findings will provide a scientific basis for developing effective intervention strategies, which may benefit more individuals and improve public health in the future. Your participation is entirely voluntary. This study has been approved by the Ethics Review Committee. If you agree to take part, please read the following information carefully.

1. You are requested to complete the questionnaire. There are no right or wrong answers; please respond based on your actual situation. You are welcome to consult us at any point during the process. Once complete, please submit the questionnaire promptly.

2. This study involves a simple questionnaire survey and poses no risk to your physical or psychological well-being. However, some items may involve personal information such as your gender and age. All responses will be kept strictly confidential and will not be disclosed. Please feel free to answer honestly.

3. As a participant, you have the right to be informed about the study and its progress at any time. If you choose to withdraw from the study, please notify us, and your data will not be included in the final analysis.

We sincerely thank you for taking the time to support our research efforts.

☐ I have read and understood the above and agree that the data collected may be used for scientific research.

Signature of Informed Consent:

Date of Participation:    Year   Month   Day

| **Section I: Basic Information** | | |
| --- | --- | --- |
| **1. Sex** | a. Male  b. Female | |
| **2. Your age:___ years** | | |
| **3. Place of residence** | a. Rural  b. Urban | |
| **4. Educational attainment** | a. Junior high school or below  b. Senior high school / Vocational secondary school  c. Junior college  d. bachelor’s degree  e. Master’s degree or above | |
| **5. Marital status** | a. Single b. Married c. Divorced d. Widowed | |
| **6. Relationship to the child** | a. Parent  b. Grandparent  c. Other | |
| **7. Monthly household income (RMB)** | a.<2000  b.2000-5000  c.5000-10000  d.10000-20000  e.>20000 | |
| **8. Have you ever been diagnosed with Mycoplasma pneumoniae pneumonia?** | a. Yes  b. No | |
| **9. Have you ever received health education or information on Mycoplasma pneumoniae pneumonia from a hospital or other institution?** | a. Yes  b. No | |
| **10. Through which sources have you acquired information about Mycoplasma pneumoniae pneumonia?** | a. Health education from hospitals, attending physicians, or other clinicians  b. Community health campaigns  c. New media (WeChat, Weibo, TikTok, Xiaohongshu, etc.)  d. Traditional media (television, newspapers, etc.)  e. Communication with relatives, friends, or other patients | |
| **11. Sex of the child** | a. Male  b. Female | |
| **12. Age of the child:___ years** | | |
| **13.** **Date of the child's diagnosis with Mycoplasma pneumoniae pneumonia:** | | a. Less than 1 month  b. 1–3 months  c. 3-6 months  d. 6-12 months  e. More than 1 year |
| **14. Has your child ever been diagnosed with Mycoplasma pneumoniae pneumonia?** | | a. Yes  b. No |

| **Section II: Knowledge Regarding Mycoplasma pneumoniae Pneumonia in Children** | | | |  |
| --- | --- | --- | --- | --- |
| **For each of the following statements, please indicate the extent of your understanding:** | | | | |
| 1. **Mycoplasma pneumoniae pneumonia is a common respiratory infection in children.** | a. Very familiar | b. Have heard of it | c. Unfamiliar | |
| 2. **Pediatric Mycoplasma pneumoniae pneumonia is caused by infection with Mycoplasma pneumoniae.** | a. Very familiar | b. Have heard of it | c. Unfamiliar | |
| 3. **Mycoplasma pneumoniae pneumonia is contagious and is primarily transmitted via airborne droplets and direct contact.** | a. Very familiar | b. Have heard of it | c. Unfamiliar | |
| 4. **The typical clinical manifestations of pediatric Mycoplasma pneumoniae pneumonia include fever and persistent dry cough.** | a. Very familiar | b. Have heard of it | c. Unfamiliar | |
| 5. **Pulmonary complications of Mycoplasma pneumoniae pneumonia may include atelectasis, pleural effusion, and respiratory failure.** | a. Very familiar | b. Have heard of it | c. Unfamiliar | |
| 6. **Approximately one-quarter of pediatric patients with Mycoplasma pneumoniae pneumonia present with extrapulmonary manifestations, such as myocarditis, arrhythmia, encephalitis, or nephropathy.** | a. Very familiar | b. Have heard of it | c. Unfamiliar | |
| 7. **The key to treatment of pediatric Mycoplasma pneumoniae pneumonia is the appropriate use of antimicrobial agents.** | a. Very familiar | b. Have heard of it | c. Unfamiliar | |
| 8. **Mycoplasma pneumoniae pneumonia is a self-limiting illness; most children have a favorable prognosis following standard treatment.** | a. Very familiar | b. Have heard of it | c. Unfamiliar | |
| 9. **Children diagnosed with Mycoplasma pneumoniae pneumonia should be properly isolated to prevent cross-infection and reinfection.** | a. Very familiar | b. Have heard of it | c. Unfamiliar | |
| 10. **Preventive measures for pediatric Mycoplasma pneumoniae pneumonia include frequent handwashing and avoiding contact with infected children.** | a. Very familiar | b. Have heard of it | c. Unfamiliar | |
| 11. **The capital of China is Shanghai.** | a. True | b. False | c. Unfamiliar | |

| **Section III: Attitudes Toward Mycoplasma pneumoniae Pneumonia in Children** | | | | | |
| --- | --- | --- | --- | --- | --- |
| 1. **I believe Mycoplasma pneumoniae pneumonia is a serious illness that requires prompt treatment.** | a. Strongly agree | b. Agree | c. Neutral | d. Disagree | e. Strongly disagree |
| 2. **Compared with other common types of pneumonia (e.g., bacterial or viral pneumonia), I feel more anxious when my child is diagnosed with Mycoplasma pneumoniae pneumonia.** | a. Strongly agree | b. Agree | c. Neutral | d. Disagree | e. Strongly disagree |
| 3. **I have sufficient confidence in the treatment of Mycoplasma pneumoniae pneumonia.** | a. Strongly agree | b. Agree | c. Neutral | d. Disagree | e. Strongly disagree |
| 4. **I believe that timely treatment can prevent severe complications.** | a. Strongly agree | b. Agree | c. Neutral | d. Disagree | e. Strongly disagree |
| 5. **I will follow the physician’s recommendations and complete the full course of treatment.** | a. Strongly agree | b. Agree | c. Neutral | d. Disagree | e. Strongly disagree |
| 6. **I am concerned about the use of antibiotics in the treatment of Mycoplasma pneumoniae pneumonia.** | a. Strongly agree | b. Agree | c. Neutral | d. Disagree | e. Strongly disagree |
| 7. **I prefer that my child receives treatment in a hospital rather than at home.** | a. Strongly agree | b. Agree | c. Neutral | d. Disagree | e. Strongly disagree |
| 8. **I believe that treatment for Mycoplasma pneumoniae pneumonia should be individualized.** | a. Strongly agree | b. Agree | c. Neutral | d. Disagree | e. Strongly disagree |
| 9. **I am willing to use non-pharmacological approaches to support my child’s recovery.** | a. Strongly agree | b. Agree | c. Neutral | d. Disagree | e. Strongly disagree |
| 10. **I am concerned about the potential recurrence of Mycoplasma pneumoniae pneumonia.** | a. Strongly agree | b. Agree | c. Neutral | d. Disagree | e. Strongly disagree |
| 11. **I believe that prevention is more important than treatment for Mycoplasma pneumoniae pneumonia.** | a. Strongly agree | b. Agree | c. Neutral | d. Disagree | e. Strongly disagree |
| 12. **I will educate my child to develop good personal hygiene habits.** | a. Strongly agree | b. Agree | c. Neutral | d. Disagree | e. Strongly disagree |
| 13. **I am willing to learn more about Mycoplasma pneumoniae pneumonia.** | a. Strongly agree | b. Agree | c. Neutral | d. Disagree | e. Strongly disagree |

| **Section IV: Practices Regarding Mycoplasma pneumoniae Pneumonia in Children**  Response scale: Always – nearly 100% of the time; Often – approximately ≥70% of the time; Sometimes – approximately 40–70% of the time; Rarely – approximately 10–40% of the time; Never – almost never or <10% of the time | | | | | |
| --- | --- | --- | --- | --- | --- |
| 1. **When my child develops symptoms such as cough and fever, I seek medical attention immediately.** | a. Always | b. Often | c. Sometimes | d. Rarely | e. Never |
| 2. **I administer medications to my child on time and as prescribed by the physician.** | a. Always | b. Often | c. Sometimes | d. Rarely | e. Never |
| 3. **I closely monitor my child’s body temperature and take appropriate measures as needed.** | a. Always | b. Often | c. Sometimes | d. Rarely | e. Never |
| 4. **During illness, I ensure that my child rests adequately and avoids strenuous activity.** | a. Always | b. Often | c. Sometimes | d. Rarely | e. Never |
| 5. **I pay attention to my child’s diet and ensure sufficient nutritional intake.** | a. Always | b. Often | c. Sometimes | d. Rarely | e. Never |
| 6. **While my child is ill, I avoid allowing contact with other children.** | a. Always | b. Often | c. Sometimes | d. Rarely | e. Never |
| 7. **I regularly clean and disinfect the household environment to prevent reinfection with Mycoplasma pneumoniae.** | a. Always | b. Often | c. Sometimes | d. Rarely | e. Never |
| 8. **After recovery, I take my child for follow-up evaluation to ensure complete resolution.** | a. Always | b. Often | c. Sometimes | d. Rarely | e. Never |
| 9. **I consult with physicians to obtain further information about Mycoplasma pneumoniae pneumonia.** | a. Always | b. Often | c. Sometimes | d. Rarely | e. Never |

**Thank you once again for participating in our questionnaire. The information you have provided is of great value to our ongoing research and future efforts.**

If you have any comments or suggestions regarding this survey, we would be grateful to hear your feedback.

Comments and suggestions:    (Optional)

To ensure that this survey contributes meaningfully to future follow-up efforts, we would sincerely appreciate it if you are willing to provide your contact information.

Your telephone number:    (Optional)
