# Supplementary material for: Knowledge, attitudes, and practices regarding mycoplasma pneumoniae pneumonia among family members of affected children
Source: Sci Rep. 2026 May 29;16:24617. doi: 10.1038/s41598-026-55543-4 (PMC13454310; doi:10.1038/s41598-026-55543-4)
Supplement: Supplementary file 2 [file 41598_2026_55543_MOESM2_ESM.docx]

**Table S1. CFA model fit indices.**

| **Indicators** | **Reference** | **Actual** |
| --- | --- | --- |
| **CMIN/DF** | **1-3: Excellent, 3-5: Good** | **2.334** |
| **RMSEA** | **<0.08: Good** | **0.055** |
| **IFI** | **>0.8: Good** | **0.927** |
| **TLI** | **>0.8: Good** | **0.918** |
| **CFI** | **>0.8: Good** | **0.926** |

**KMO=0.919 (P<0.001).**

**Table S2. CFA factor loadings for the KAP measurement model.**

|  |  |  | **Estimate** | **Standardized Estimate** | **S.E.** | **C.R.** | **P** |
| --- | --- | --- | --- | --- | --- | --- | --- |
| **K1** | **<---** | **Knowledge** | **1.000** | **0.760** |  |  |  |
| **K2** | **<---** | **Knowledge** | **1.174** | **0.786** | **0.067** | **17.442** | **<0.001** |
| **K3** | **<---** | **Knowledge** | **1.247** | **0.827** | **0.068** | **18.370** | **<0.001** |
| **K4** | **<---** | **Knowledge** | **1.156** | **0.812** | **0.064** | **18.123** | **<0.001** |
| **K5** | **<---** | **Knowledge** | **1.130** | **0.682** | **0.076** | **14.820** | **<0.001** |
| **K6** | **<---** | **Knowledge** | **1.081** | **0.649** | **0.077** | **14.004** | **<0.001** |
| **K7** | **<---** | **Knowledge** | **1.149** | **0.771** | **0.067** | **17.052** | **<0.001** |
| **K8** | **<---** | **Knowledge** | **1.087** | **0.711** | **0.071** | **15.373** | **<0.001** |
| **K9** | **<---** | **Knowledge** | **1.124** | **0.766** | **0.066** | **16.909** | **<0.001** |
| **K10** | **<---** | **Knowledge** | **1.008** | **0.728** | **0.063** | **15.940** | **<0.001** |
| **A1** | **<---** | **Attitude** | **1.000** | **0.554** |  |  |  |
| **A2** | **<---** | **Attitude** | **0.952** | **0.508** | **0.088** | **10.763** | **<0.001** |
| **A3** | **<---** | **Attitude** | **1.056** | **0.673** | **0.099** | **10.678** | **<0.001** |
| **A4** | **<---** | **Attitude** | **1.078** | **0.788** | **0.092** | **11.666** | **<0.001** |
| **A5** | **<---** | **Attitude** | **1.057** | **0.797** | **0.090** | **11.744** | **<0.001** |
| **A6** | **<---** | **Attitude** | **0.448** | **0.188** | **0.122** | **3.668** | **<0.001** |
| **A7** | **<---** | **Attitude** | **1.186** | **0.630** | **0.116** | **10.236** | **<0.001** |
| **A8** | **<---** | **Attitude** | **1.065** | **0.619** | **0.105** | **10.117** | **<0.001** |
| **A9** | **<---** | **Attitude** | **0.784** | **0.320** | **0.130** | **6.009** | **<0.001** |
| **A10** | **<---** | **Attitude** | **0.940** | **0.541** | **0.102** | **9.222** | **<0.001** |
| **A11** | **<---** | **Attitude** | **1.017** | **0.647** | **0.098** | **10.403** | **<0.001** |
| **A12** | **<---** | **Attitude** | **0.968** | **0.766** | **0.084** | **11.488** | **<0.001** |
| **A13** | **<---** | **Attitude** | **1.092** | **0.788** | **0.093** | **11.683** | **<0.001** |
| **P1** | **<---** | **Practice** | **1.000** | **0.390** |  |  |  |
| **P2** | **<---** | **Practice** | **0.977** | **0.583** | **0.117** | **8.373** | **<0.001** |
| **P3** | **<---** | **Practice** | **1.310** | **0.767** | **0.165** | **7.920** | **<0.001** |
| **P4** | **<---** | **Practice** | **1.420** | **0.815** | **0.177** | **8.039** | **<0.001** |
| **P5** | **<---** | **Practice** | **1.537** | **0.820** | **0.191** | **8.050** | **<0.001** |
| **P6** | **<---** | **Practice** | **1.593** | **0.753** | **0.202** | **7.881** | **<0.001** |
| **P7** | **<---** | **Practice** | **1.276** | **0.507** | **0.187** | **6.828** | **<0.001** |
| **P8** | **<---** | **Practice** | **1.367** | **0.475** | **0.206** | **6.623** | **<0.001** |
| **P9** | **<---** | **Practice** | **1.422** | **0.553** | **0.200** | **7.095** | **<0.001** |

**Table S3. Distribution of responses in the knowledge dimension (N=449)**

| **Knowledge items, n (%)** | **Very familiar** | **Heard of it** | **Unclear** |
| --- | --- | --- | --- |
| 1.**Mycoplasma pneumonia is a common respiratory tract infection in children.** | 164(36.53) | 262(58.35) | 23(5.12) |
| 2.**Pediatric mycoplasma pneumonia is caused by infection with Mycoplasma pneumoniae.** | 168(37.42) | 233(51.89) | 48(10.69) |
| 3.**Mycoplasma pneumonia is contagious and is mainly transmitted through airborne droplets and direct contact.** | 198(44.1) | 208(46.33) | 43(9.58) |
| 4.**Typical symptoms of pediatric mycoplasma pneumonia include fever and persistent dry cough** | 198(44.1) | 220(49) | 31(6.9) |
| 5.**Pulmonary complications of mycoplasma pneumonia may include atelectasis, pleural effusion, and respiratory failure.** | 123(27.39) | 222(49.44) | 104(23.16) |
| 6.**About one-quarter of children with mycoplasma pneumonia have extrapulmonary manifestations, which may include myocarditis, arrhythmia, encephalitis, and kidney disease.** | 103(22.94) | 219(48.78) | 127(28.29) |
| 7.**The key to treating pediatric mycoplasma pneumonia is selecting appropriate antibiotics.** | 160(35.63) | 239(53.23) | 50(11.14) |
| 8.**Mycoplasma pneumonia is a self-limiting disease, and most children have a good prognosis after proper treatment.** | 151(33.63) | 238(53.01) | 60(13.36) |
| 9.**Children with mycoplasma pneumonia should be isolated to prevent cross-infection and reinfection.** | 213(47.44) | 201(44.77) | 35(7.8) |
| 10.**Preventive measures for pediatric mycoplasma pneumonia mainly include frequent handwashing and avoiding contact with infected children.** | 236(52.56) | 190(42.32) | 23(5.12) |

**Table S4. Distribution of responses in the attitude dimension (N=449)**

| **Attitude items, n (%)** | **Strongly agree** | **Agree** | **Neutral** | **Disagree** | **Strongly disagree** |
| --- | --- | --- | --- | --- | --- |
| 1.**I believe mycoplasma pneumonia is a serious illness that requires timely treatment.** | 224(49.89) | 179(39.87) | 37(8.24) | 9(2) | / |
| 2.**Compared to other common types of pneumonia (such as bacterial or viral pneumonia), I feel more anxious when my child has mycoplasma pneumonia.** | 152(33.85) | 191(42.54) | 105(23.39) | 1(0.22) | / |
| 3.**I have sufficient confidence in the treatment of mycoplasma pneumonia.** | 187(41.65) | 228(50.78) | 32(7.13) | 2(0.45) | / |
| 4.**I believe timely treatment can prevent severe complications.** | 267(59.47) | 174(38.75) | 6(1.34) | 2(0.45) | / |
| 5.**I will follow the doctor’s advice and complete the full course of treatment.** | 277(61.69) | 165(36.75) | 6(1.34) | 1(0.22) | / |
| 6.**I am concerned about the use of antibiotics for treating mycoplasma pneumonia.** | 97(21.6) | 167(37.19) | 136(30.29) | 42(9.35) | 7(1.56) |
| 7.**I prefer my child to receive treatment in a hospital rather than at home.** | 201(44.77) | 191(42.54) | 47(10.47) | 9(2) | 1(0.22) |
| 8.**I believe the treatment of mycoplasma pneumonia should be individualized.** | 178(39.64) | 217(48.33) | 50(11.14) | 4(0.89) | / |
| 9.**I am willing to use non-pharmacological methods to help my child recover.** | 130(28.95) | 181(40.31) | 88(19.6) | 46(10.24) | 4(0.89) |
| 10.**I worry that mycoplasma pneumonia might recur.** | 162(36.08) | 238(53.01) | 41(9.13) | 7(1.56) | 1(0.22) |
| 11.**I believe prevention is more important than treatment for mycoplasma pneumonia.** | 228(50.78) | 194(43.21) | 24(5.35) | 3(0.67) | / |
| 12.**I will educate my child to develop good personal hygiene habits.** | 300(66.82) | 144(32.07) | 4(0.89) | 1(0.22) |  |
| 13.**I am willing to learn more about mycoplasma pneumonia.** | 272(60.58) | 167(37.19) | 8(1.78) | 2(0.45) |  |

**Table S5. Distribution of responses in the practice dimension (N=449)**

| **Practice items, n (%)** | **Always** | **Often** | **Sometimes** | **Rarely** | **Never** |
| --- | --- | --- | --- | --- | --- |
| **1. When my child shows symptoms of cough and fever, I will take them to see a doctor immediately.** | 182(40.53) | 170(37.86) | 77(17.15) | 19(4.23) | 1(0.22) |
| **2. I will give my child the medication on time as instructed by the doctor.** | 311(69.27) | 126(28.06) | 9(2) | 2(0.45) | 1(0.22) |
| **3. I will closely monitor my child’s body temperature and take prompt action when necessary.** | 329(73.27) | 105(23.39) | 11(2.45) | 3(0.67) | 1(0.22) |
| **4. During the illness, I will make sure my child gets enough rest and avoids strenuous activities.** | 317(70.6) | 117(26.06) | 12(2.67) | 1(0.22) | 2(0.45) |
| **5. I will pay attention to my child’s diet to ensure they get adequate nutrition.** | 293(65.26) | 133(29.62) | 20(4.45) | 1(0.22) | 2(0.45) |
| **6. While my child is ill, I will prevent them from coming into contact with other children.** | 283(63.03) | 133(29.62) | 25(5.57) | 5(1.11) | 3(0.67) |
| **7. I will regularly clean and disinfect the home environment to prevent reinfection with mycoplasma pneumonia.** | 208(46.33) | 166(36.97) | 59(13.14) | 12(2.67) | 4(0.89) |
| **8. After my child recovers, I will take them for a follow-up check to ensure complete recovery.** | 203(45.21) | 141(31.4) | 73(16.26) | 27(6.01) | 5(1.11) |
| **9. I will consult the doctor for more information about mycoplasma pneumonia.** | 217(48.33) | 156(34.74) | 53(11.8) | 22(4.9) | 1(0.22) |

**Table S6. Structural equation model fit indices**

| Indicators | Reference | Results |
| --- | --- | --- |
| RMSEA | <0.08 Good | 0.051 |
| SRMR | <0.08 Good | 0.052 |
| TLI | >0.8 Good | 0.928 |
| CFI | >0.8 Good | 0.937 |
