# Supplementary material for: Knowledge, attitudes, and practices regarding mycoplasma pneumoniae pneumonia among family members of affected children
Source: Sci Rep. 2026 May 29;16:24617. doi: 10.1038/s41598-026-55543-4 (PMC13454310; doi:10.1038/s41598-026-55543-4)
Supplement: Supplementary file 3 [file 41598_2026_55543_MOESM3_ESM.docx]

**Figure S1. Flowchart of participant selection for the KAP survey**
